# Mesenchymal Stem Cells Derived from Human Exfoliated Deciduous Teeth (SHEDs) Induce Immune Modulatory Profile in Monocyte-Derived Dendritic Cells

**DOI:** 10.1371/journal.pone.0098050

**Published:** 2014-05-20

**Authors:** Fernando de Sá Silva, Rodrigo Nalio Ramos, Danilo Candido de Almeida, Enio Jose Bassi, Roberto Pereira Gonzales, Sueli Patricia Harumi Miyagi, Claudinéia Pereira Maranduba, Osvaldo Augusto Brazil Esteves Sant'Anna, Márcia Martins Marques, José Alexandre Marzagão Barbuto, Niels Olsen Saraiva Câmara, Carlos Magno da Costa Maranduba

**Affiliations:** 1 Biomedical Science Institute, University of São Paulo, São Paulo, Brazil; 2 Dentistry Department, University of São Paulo, São Paulo, Brazil; 3 Immunochemistry Laboratory, Butantan Institute, São Paulo, Brazil; 4 Biomedical Science Institute, University of Juiz de Fora, Juiz de Fora, Brazil; Istituto Superiore di Sanità, Italy

## Abstract

**Background:**

Mesenchymal stem cells have prominent immune modulatory properties, which may have clinical applications; however their major source, bone marrow, is of limited availability. On the other hand, mesenchymal stem cells derived from human exfoliated deciduous teeth (SHEDs) are readily accessible, but their immune regulatory properties have not been completely investigated. This study was designed, therefore, to evaluate the SHEDs influence on DCs differentiation, maturation, ability to activate T cells and to expand CD4^+^Foxp3^+^ T cells.

**Methodology/Principal Findings:**

The experiments were based in cellular co-culture during differentiation and maturation of monocyte derived-DCs (moDCs), with, or not, presence of SHEDs. After co-culture with SHEDs, (moDCs) presented lower expression of BDCA-1 and CD11c, in comparison to DC cultivated without SHEDs. CD40, CD80, CD83 and CD86 levels were also decreased in mature DCs (mDCs) after co-cultivation with SHEDs. To assess the ability of SHEDs-exposed moDCs to modulate T cell responses, the former were separated from SHEDs, and co-cultured with peripheral blood lymphocytes. After 5 days, the proliferation of CD4^+^ and CD8^+^ T cells was evaluated and found to be lower than that induced by moDCs cultivated without SHEDs. In addition, an increase in the proportion of CD4^+^Foxp3^+^IL-10^+^ T cells was observed among cells stimulated by mature moDCs that were previously cultivated with SHEDs. Soluble factors released during co-cultures also showed a reduction in the pro-inflammatory cytokines (IL-2, TNF-α and IFN-γ), and an increase in the anti-inflammatory molecule IL-10.

**Conclusion/Significance:**

This study shows that SHEDs induce an immune regulatory phenotype in moDCs cells, evidenced by changes in maturation and differentiation rates, inhibition of lymphocyte stimulation and ability to expand CD4^+^Foxp3^+^ T cells. Further characterization and validation of this phenomenon could support the use of SHEDs, directly or indirectly for immune modulation in the clinical practice.

## Introduction

Mesenchymal stem cells (MSCs) are multipotent stromal adult stem cells able to differentiate into mesodermal lineages (osteocytes, adipocytes and chondrocytes) *in vitro,* possessing prominent regulatory properties on innate and adaptive immune responses. The MSCs can exert their immune suppressive potential by cell-to-cell contact and/or by secretion of immune regulatory molecules, such as IDO, TGF-β and PGE2 [1]–[6].

Human MSCs can suppress T cell proliferation [1], [3], [7], inhibit cell lysis promoted by cytotoxic CD8^+^ T lymphocytes and by natural killer cells (Rasmusson et al., 2003), besides having the ability to reduce pro-inflammatory and increase anti-inflammatory factors production [5], [6], [8]. Moreover, MSCs are not immunogenic, since these cells do not express major histocompatibility complex class II (MHC II), independently of their source (autologous or allogeneic) or their differentiation status [3].

MSCs can be derived from several tissues (adult and fetal) with the bone marrow and the adipose tissue as the major sources [9]. The dental pulp represents an interesting and accessible alternative source for the isolation of MSCs [10]. Indeed, the dental pulp stem cells (DPSCs) have the same bone marrow MSCs phenotype, including their immune regulatory potential. Pierdomenico et al. (2005) compared the immune modulatory capacity of bone marrow MSCs (BMSCs) and DPSC by co-cultivating these cells with CD2^+^ T cells. In that study, the authors observed that DPSC were able to reduce T cell proliferation more intensely than BMSC [11]. In addition, another study using DPSC from human exfoliated deciduous teeth (SHEDs) demonstrated that these cells inhibited Th17 cell proliferation more efficiently than BMSCs [12]. However, Alipour et al. (2013) showed that BMSCs have a greater ability to inhibit T cells proliferation compared with SHEDs [13]. These results show lack of studies and more evidences for better understanding of immune modulation by DPSCs or SHEDs.

Furthermore, the immune suppressive properties of SHEDs were demonstrated *in vivo* in an experimental mouse model of systemic lupus erythematosus (SLE). In this case, SHEDs were able to attenuate disease symptoms, decreasing the levels of autoantibodies, serum creatinine and proteinuria index [12].

Dendritic cells (DCs) are of great importance in the general context of early immune responses, since these cells are the main antigen-presenting cells (APCs). They are major players in onset of immune responses; they affect significantly the balance between helper and regulatory T cells; they establish tolerance to self-antigens; and have a definite role in transplantation settings [5], [12]–[14].

The immune regulatory potential of MSCs on DCs, however, remains incompletely explored. Zhang et al. (2004) observed in co-cultures between BMSCs and DCs that BMSCs inhibited the expression of several DCs maturation markers (CD40, CD83 and CD86), decreasing their ability to activate T cells responses [4]. Aggarwal et al. (2005) showed that BMSCs can lead DCs to favor an immunological tolerance state, due to the inhibition of TNF-α and increase in IL-10 production [5]. Finally, Lai et al. (2010) observed that BMSCs in co-culture with hematopoietic stem cells (HSCs) inhibited the generation of myeloid DCs [14].

Here we attempt to characterize the impact of SHEDs upon the differentiation and maturation of moDCs. For this, we evaluated the effects of SHEDs presence during their differentiation on the surface phenotype of moDCs and the ability of the latter to activate/inhibit T cells, determining also, specifically, the response of the CD4^+^Foxp3^+^ T cell population.

## Materials and Methods

### Ethics Statement

Dental pulp and peripheral blood were obtained from healthy volunteers after written informed consent and approval by the Institutional Review Board at Dentistry School/University of São Paulo (number 129/10).

### Cell Isolation and Culture

SHEDs isolation and culture were performed as previously described by Gronthos et al. (2000) and Oliveira et al. (2009) [10], [15]. The cells were cultivated with basal medium, consisting of F12 medium (Gibco, Grand Island, NY, USA) supplemented with 15% serum Hyclone (Thermo, Logan, UT, USA), antibiotic-antimycotic solution (100 U/ml penicillin, 100 µg/ml streptomycin, and 25 µg/ml amphotericin; Gibco), 2.5 mM L-glutamine and nonessential amino acids (Gibco). To obtain DCs, peripheral blood mononuclear cells (PBMCs) were obtained using Ficoll-paque (Amersham Pharmacia Biotech, Uppsala, Sweden). PBMCs were resuspended and seeded in six well plates containing RPMI-1640 culture medium (Gibco), supplemented with 10% FCS (Gibco) and antibiotic-antimycotic solution. After two hours of incubation at 37°C in 5% CO2, non-adherent cells were removed. To obtain immature DCs (iDCs), adhered monocytes were cultured, alone or with SHEDs, in presence of GM-CSF and IL-4 (50 ng/mL; Peprotech, Rocky Hill, NJ, USA). To generate mature DCs (mDCs) bacterial lipopolysaccharide (LPS, 50 µg/mL; *Escherichia coli* 0111:B4; Sigma-Aldrich, St. Louis, MO, USA) was added to the culture on day five [16]. Non-adherent cells were used as responding cells in allogeneic mixed DC-T cell cultures.

### Phenotype and Differentiation Capacity of SHEDs

To verify SHEDs phenotype, the cells were exposed to two conditions: culture in basal medium or cultured in moDCs maturation medium (seven days in presence of IL-4 and GM-CSF, and the last two days in presence of LPS). After culture, the cells were harvested by treatment with trypsin (Gibco), washed and suspended in phosphate-buffered saline (PBS); approximately 1×10^5^ cells were incubated on ice for 20 minutes with conjugated monoclonal antibodies (1∶100) against CD73, CD90, CD105, CD45, CD34, CD11c, CD14, CD40, CD80, CD83, CD86, HLA-DR (BD Biosciences, San Jose, CA, USA) and BDCA-1 (Miltenyi Biotec, Germany). The acquisition was done in a FACSCanto II flow cytometer (BD Biosciences) and the analysis performed using the FlowJo software, Ver.7.2.4 (Tree Star, Ashland, OR, USA).

SHEDs adipogenic and osteogenic differentiation was evaluated, as described by Pittenger et al. (1999) [17]. SHEDs were cultivated until total confluence and, then, induced to differentiation by the Mesenchymal Stem Cell Adipogenesis Kit and Mesenchymal Stem Cell Osteogenesis Kit respectively (Chemicon, Temecula, CA, USA), according to the manufacturer instructions. The medium was replaced every three to four days over a period of 21 days. Adipogenesis was determined by staining with oil red O, to verify neutral lipids accumulation in fat vacuoles, and osteogenesis, by detection of calcium compounds (by Alizarin Red staining).

### SHEDs and DCs Coculture

To determine SHEDs effects on moDCs differentiation and maturation, SHEDs and monocytes, isolated from unrelated donors, were co-cultured (SHEDs to monocytes ratio, 1∶10; 1∶20; 1∶100; for 2×10^5^ monocytes) in the presence of GM-CSF and IL-4 for seven days, to obtain immature DCs (iDCs), and with LPS, in the last 48 hours, to obtain mature DCs (mDCs) [4], [5], [16], [18]–[20]. SHEDs were also added to already differentiated immature moDCs (at day 5 of monocyte culture in the presence of cytokines). All cells were harvested on day seven, labeled with the various specific fluorescent antibodies (CD11c, CD14, CD40, CD80, CD83, CD86 and HLA-DR; BD Biosciences; and BDCA; Miltenyi Biotec) and acquired by FACSCanto II flow cytometer (BD Biosciences). Co-culture supernatants were harvested for cytokine quantification assays. To exclude SHEDs from moDCs analysis, gates for typical FSC and SSC, doublet exclusion and HLA-DR^+^CD11c^+^ cells were used. The results obtained are presented as an index of Relative Fluorescence Intensity (RFI), which was calculated by the ratio between median fluorescence intensity (MFI) from experimental groups and MFI from control group (iDCs without SHEDs).

### MoDCs Isolation from Co-cultures

MoDCs functional analyses were performed to assess the ability of moDCs (previously co-cultured, or not, with SHEDs) to stimulate T cell responses [16], [20]. Since SHEDs do not express HLA-DR, MoDCs were harvested by the isolation of HLA-DR^+^ cells using immunomagnetic selection beads according to the manufacturer’s instructions (Miltenyi Biotech). The isolated cell population was more than 90% positive for CD45.

### DCs and T cells Co-cultures

Non-adherent PBMC, harvested after two hours of culture, were labeled with 5 µM carboxyfluorescein succinimidyl ester (CFSE, BD Bioscience). iDCs or mDCs from non-related donors (previously co-cultured, or not, with SHEDs) were co-cultured with 1×10^5^ non-adherent cells in a 1∶10 ratio. After five days, T cells were stained with anti-CD4 and anti-CD8 antibodies (BD Biosciences) and CFSE dilution was determined by flow cytometry (FACSCanto II cytometer, BD Biosciences). This analysis was performed within the gates of interest (lymphocytes’ FSC and SSC, CD4^+^ or CD8^+^ cells) and, at least, 20,000 events were acquired. Phytohemagglutinin A (PHA) was used as a polyclonal-positive stimulus. Co-culture supernatants were also collected for cytokine quantification assay.

### Intracellular Staining

To investigate the profile of stimulated T cells, intracellular FoxP3, IL-10 and IFN-γ expression were determined at the end of co-culture with moDCs. Cell permeabilization and fixation were made using the Fix/Perm Buffer Set kit (Biolegend, EUA). Two sets of labeling were used: FoxP3 (Alexa 488), CD4 (PE) e IL-10 (APC) and FoxP3 (Alexa 488), CD4 (APC) and IFN-γ (PE), all at 1∶100 dilution (BD Biosciences and Biolegend, USA). Analyses were performed in a FACSCanto II flow cytometer.

### Cytokine Quantification Assay

Supernatants from moDCs cultures (control), and SHEDs:moDCs and T cells:moDCs co-cultures were harvested; and their cytokine content determined by BD Cytometric Bead Array to Human (CBA) Th1/Th2/Th17 Kit to detect IL-2, IL-4, IL-6, IL-10, TNF, IFN-γ, and IL-17A according to the manufacturers’ instructions (BD Biosciences). The acquisition was done in the FACSCanto II cytometer (BD Biosciences) and the data analyzed using FCAP Array Software v3.0 (Soft Flow Inc.).

### Statistical Analysis

Results were checked for normality by the Kolmogorov-Smirnov test. moDCs’ expression of differentiation and maturation markers was evaluated using the Kruskal-Wallis test and Dunn’s post-test. T cell proliferation and expression of Foxp3, IFN-γ and IL-10, as well as cytokines assays were evaluated using the Student’s t-test. Two-tail analyses were used in all tests and the different values of p, were represented as follows: *p<0.05; **p<0.01; ***p<0.001.

## Results

### SHEDs Express MSC-related Markers and have Multipotent Differentiation Potential

**Figure 1:**
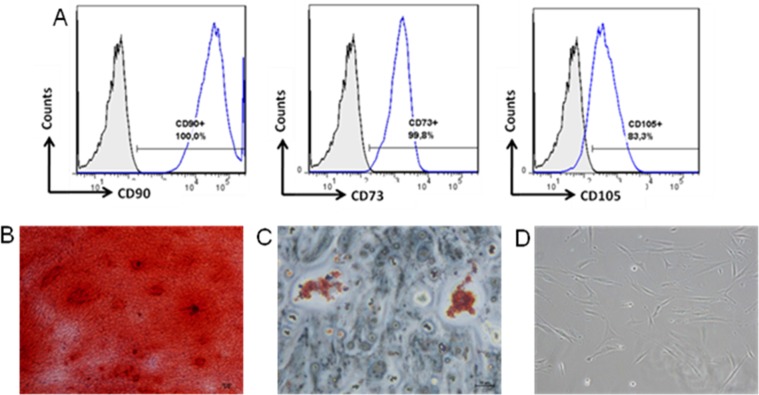
SHEDs express characteristic of MSCs as predicted markers and differentiation potential into mesodermal lineages. (A) SHEDs was characterized by expression of mesenchymal markers CD90, CD73 and CD105. For prove stem cell plasticity of SHEDs, this cell was differentiated to: osteogenic lineage and Alizarin Red was used for stained mineral deposition (B); adipogenic lineage analyzed by neutral lipids accumulation in fat vacuoles stained with oil red O (C); and undifferentiated SHEDs fibroblast-like morphology without differentiation stimulus (D).

SHEDs share many features of bone marrow-derived MSCs such as fibroblast-like morphology (Figure 1D) and surface markers expression. The cells were positive for CD73, CD90 and CD105 and negative for hematopoietic markers such as CD34 and CD45 (data not shown) (Figure 1A). Moreover, SHEDs demonstrated mesenchymal differentiation potential, as determined by their osteogenic and adipogenic differentiation (Figure 1B and 1C).

**Figure 2:**
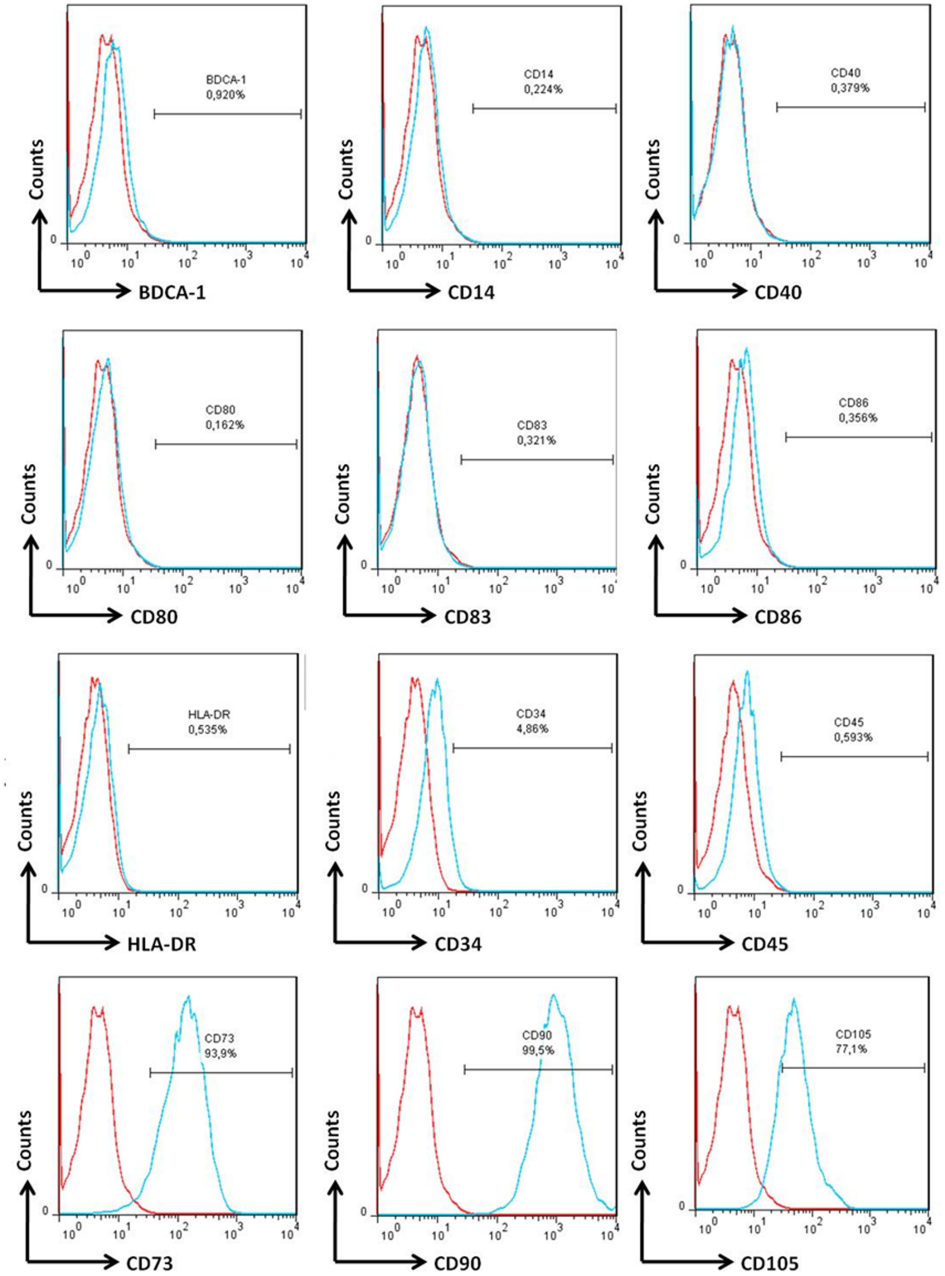
SHEDs with Mo-DCs differentiation/activation medium do not change their intrinsic features. SHEDs were cultured with medium for moDCs differentiation/activation to verified the possibility of SHEDs alter their characteristics. MoDCs differentiation medium was supplemented with IL-4 and GM-CSF for a total of seven days and at last two days received LPS. SHEDs maintained their MSCs phenotype with negative expression of hematopoietic (CD34), leukocytic (CD45) and myeloid lineage (CD14) and also to moDCs markers (BDCA-1) as well as HLA-DR.

Since SHEDs would be cultured in the DCs differentiation/activation medium (R10 supplemented with IL-4, GM-CFS and LPS), it was necessary to test the possible interference of this cocktail medium on the surface phenotype of SHEDs. Therefore, after 7 days of culture in this medium, SHEDs were harvested and analyzed by flow cytometry. The phenotype of SHEDs did not change; they remained negative for BDCA-1 and CD11c, and did not express the co-stimulatory molecules CD80, CD83, CD86 and CD40 (Figure 2). SHEDs continued negative for CD34 and CD45 (hematopoietic markers) and positive for CD73, CD90 and CD105 (mesenchymal markers). Finally, even after LPS stimulation, SHEDs did not express MHC class II molecules (Figure 2).

### Evaluation of moDCs Differentiation and Maturation in the Presence of SHEDs

**Figure 3:**
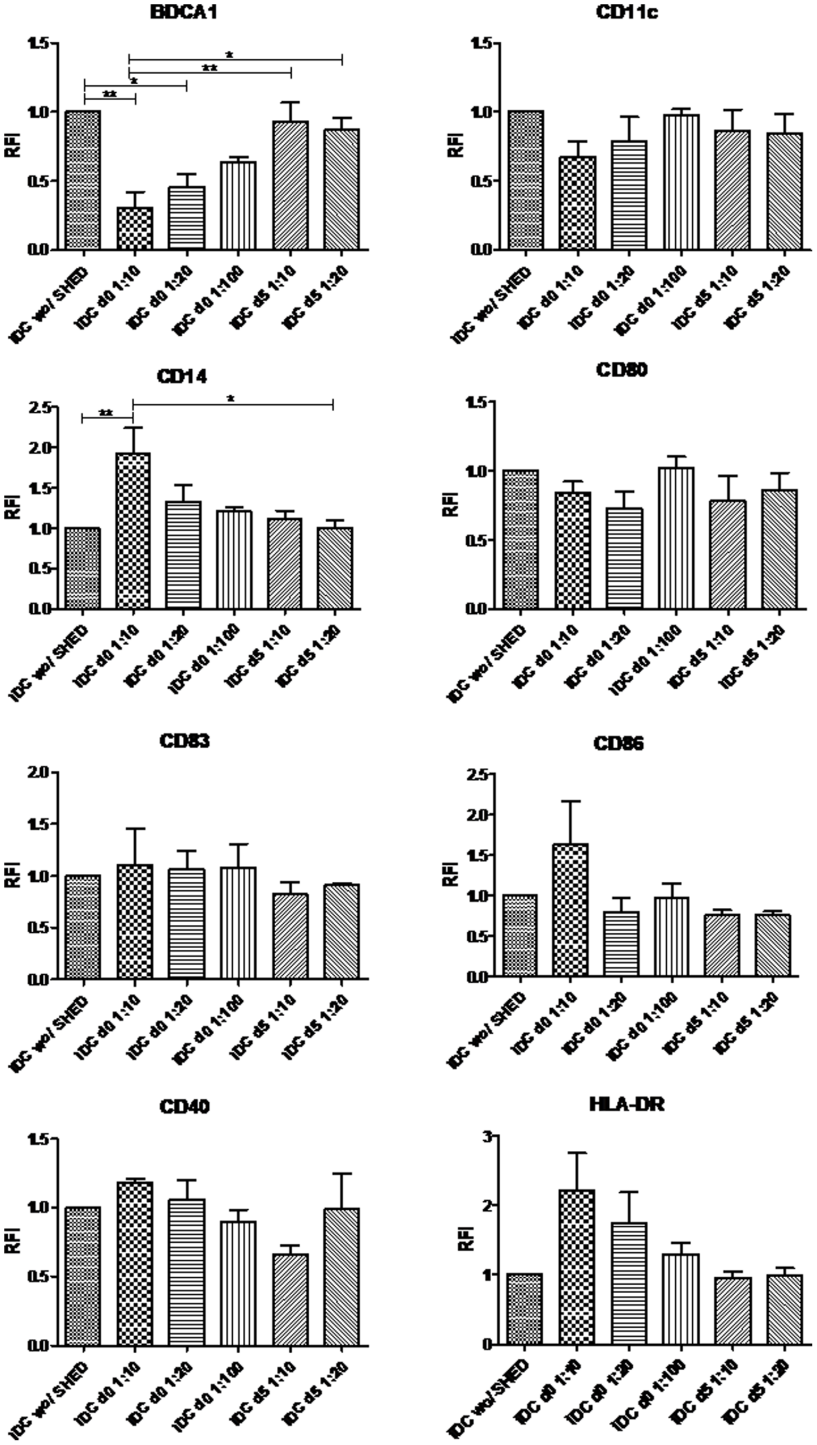
SHEDs induce dose-dependent decreasing of differentiation markers on immature moDCs. iDCs were differentiated in presence of SHEDs during seven days. The groups related to iDCs 1∶10 dilution since day zero showed an evident reduction for the BDCA-1 marker and an increase for the CD14 marker, when compared with DCs without SHEDs presence. Markers that indicate DCs maturation did not present significant RFI changes in the iDCs group, once it did not receive LPS to stimulate maturation. The characteristic molecules of them were analyzed by RFI (Relative Fluorescence Intensity). RFI presented here as a ratio between median fluorescence intensity (MFI) from experimental groups and MFI from control group (iDCs without SHEDs). iDCs – differentiated and imature DCs; d0– iDCs co-cultured with SHEDs since day zero; d5 - iDCs co-cultured with SHEDs from day five; *p≤0,05 and **p≤0,01 for the groups showed significant differences; n = 4.

SHEDs and monocytes were isolated from unrelated donors and co-cultured, either from the start or after iDCs had already been generated (day five of monocyte cultures in the presence of IL-4 and GM-CSF). When SHEDs were present since the beginning of monocyte cultures, the expression of BDCA-1 was significantly decreased, and this was proportional to the number of SHEDs added to the cultures, while the CD14 monocyte/macrophage marker increased (92.7% at the 1∶10 SHEDs concentration) (Figure 3). Analysis of CD11c expression also demonstrated a decreasing RFI index in the presence of SHEDs since the beginning of the cultures. None of the other markers analyzed in the iDC cultures achieved statistically significant changes, but, intriguingly, the expression of HLA-DR showed a dose-dependent increase in the presence of SHEDS (Figure 3). It is noteworthy that these changes were only observed when SHEDs were present since the beginning of monocytes’ differentiation, but not when the addition of SHEDs occurred at day 5, when the cells had already differentiated into iDCs, indicating that the process of moDCs differentiation was the actual target of SHEDs presence.

**Figure 4:**
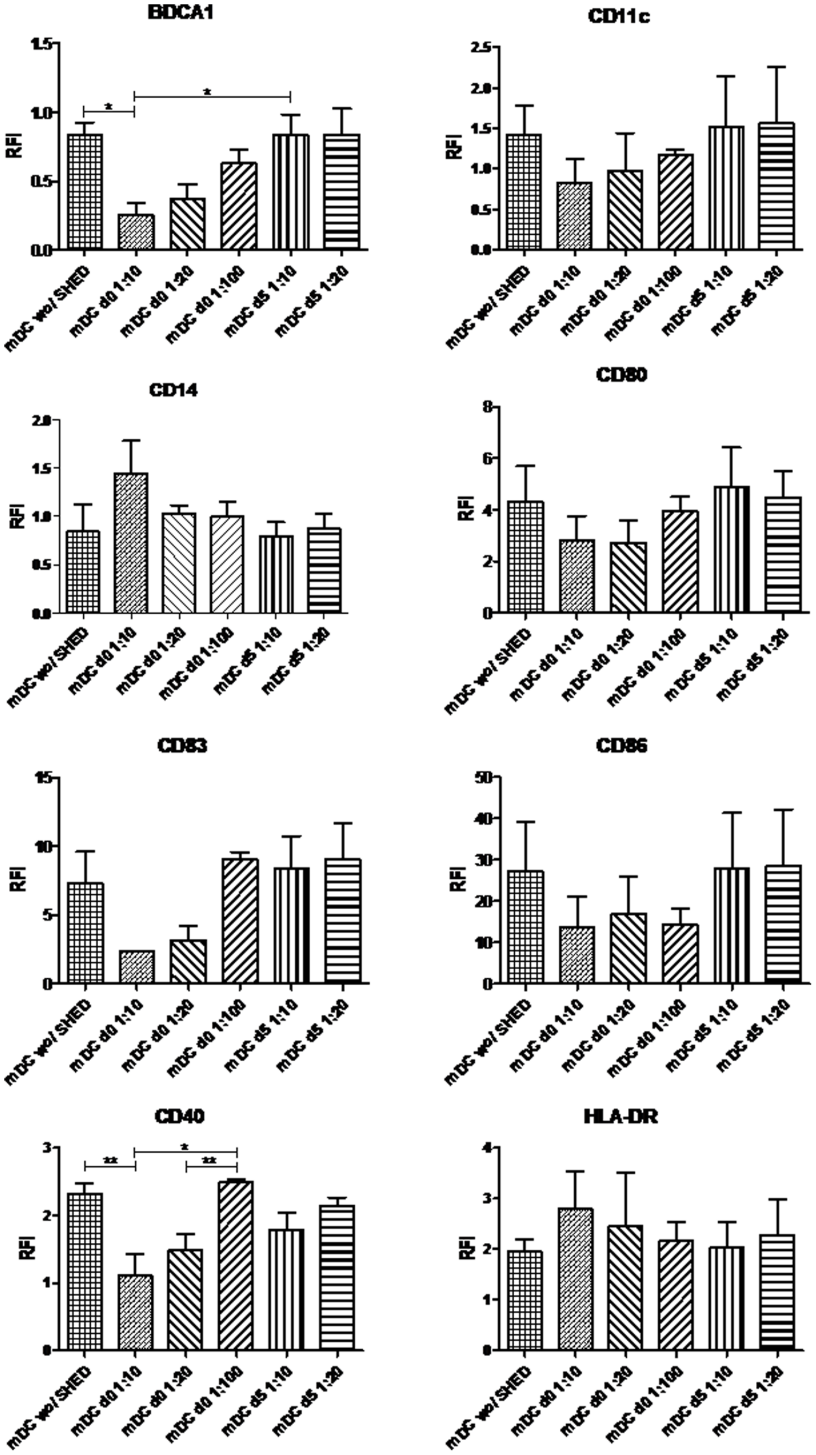
SHEDs induce ratio-dependent BDCA1 and CD40 decrease in LPS activated moDCs. During seven days under differentiation stimulus moDCs were matured in presence of SHEDs during the last two days of culture. The 1∶10 proportion group, since day zero, showed the most evident immune modulation by SHEDs on mDCs. Differentiation marker BDCA-1 was attenuated and co-stimulatory marker CD40, expressed on moDCs matured, was attenuated too. The characteristic molecules of them were analyzed by RFI (Relative Fluorescence Intensity). RFI presented here is a ratio between median fluorescence intensity (MFI) from experimental groups and MFI from control group (mDCs without SHEDs). mDCs – differentiated and mature DCs; d0– DCs co-cultured with SHEDs since day zero.; d5 - mDCs co-cultured with SHEDs from day five; *p≤0,05 and **p≤0,01 for the groups that showed significant differences; n = 4.

In order, therefore, to further characterize the effects of SHEDs upon the generation of mDCs *in vitro,* we evaluated the phenotype of moDCs exposed to SHEDs and to the maturation stimulus, LPS. Once more, the effects of SHEDs were only significant when they were added before moDC differentiation and, they were also dose-dependent, decreasing with the lower proportions of SHEDs in the cultures. Though only two of the molecules analyzed were significantly affected (BDCA-1 and CD40), the other molecules, CD11c, CD80, CD86 and CD83 also showed a tendency to decrease in the presence of SHEDs, while the expression of HLA-DR ran in the opposite direction, tending to increase with the increasing presence of SHEDs (Figure 4).

**Figure 5:**
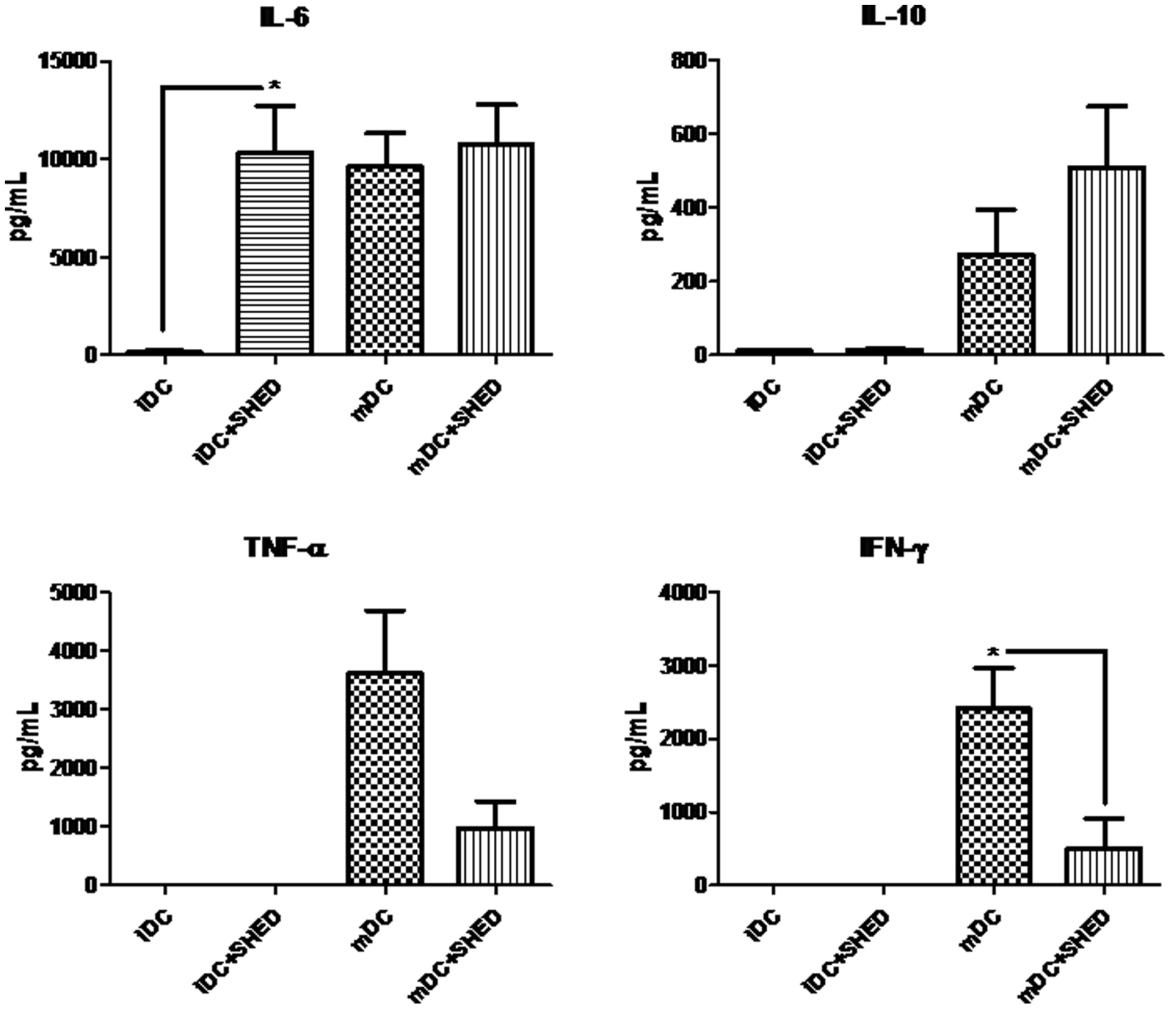
SHEDs modulate the cytokine profile in co-culture with iDCs and mDCs. Graphics indicate soluble factors levels (pg/mL; detection by CBA technique) present in the culture moDCs after seven days of differentiation (co-cultured with or without SHEDs, in a 1∶10 ratio) stimulated (to generate mDC) or not (to maintained iDC) with LPS (during the last two days of culture). Comparisons were done using t-test between iDC and iDC+SHED and between mDC and mDC+SHED; *p≤0.05 and **p≤0.01; n = 4.

To investigate the impact of SHEDs on the cytokine profile produced by DCs, their culture supernatants were analyzed. IL-6 levels in iDCs culture supernatants increased significantly (p≤0.05) in the presence of SHEDs reaching the same levels found in mDCs culture supernatants with or without SHEDs. TNF-α and IFN-γ levels were very low or undetectable in cultures of iDCs, regardless of the presence of SHEDs. However, after LPS stimulation, the level of these two factors increased in the moDCs cultures, a phenomenon that was antagonized by presence of SHEDs, with a decrease of 73% for TNF-α (non-statistically significant difference) and of 79% for IFN-γ (p≤0.05). On the other hand, IL-10 levels in mDCs culture supernatants increased, though not significantly, by 86% in the presence of SHEDs (Figure 5). IL-17 was not detected in any culture supernatant while IL-4, which was added to the cultures, did not change with the various different culture conditions (data not shown).

### MoDCs Pre-incubated by SHEDs are Limited to Induce T cells Proliferation

To assess the ability of moDCs to stimulate T cell responses after SHEDs influence, we performed a co-culture between DCs and T cells. Due the fact that SHEDs did not present HLA-DR expression, this molecule was chosen to isolate DCs cells from SHEDs after initial co-culture.

**Figure 6:**
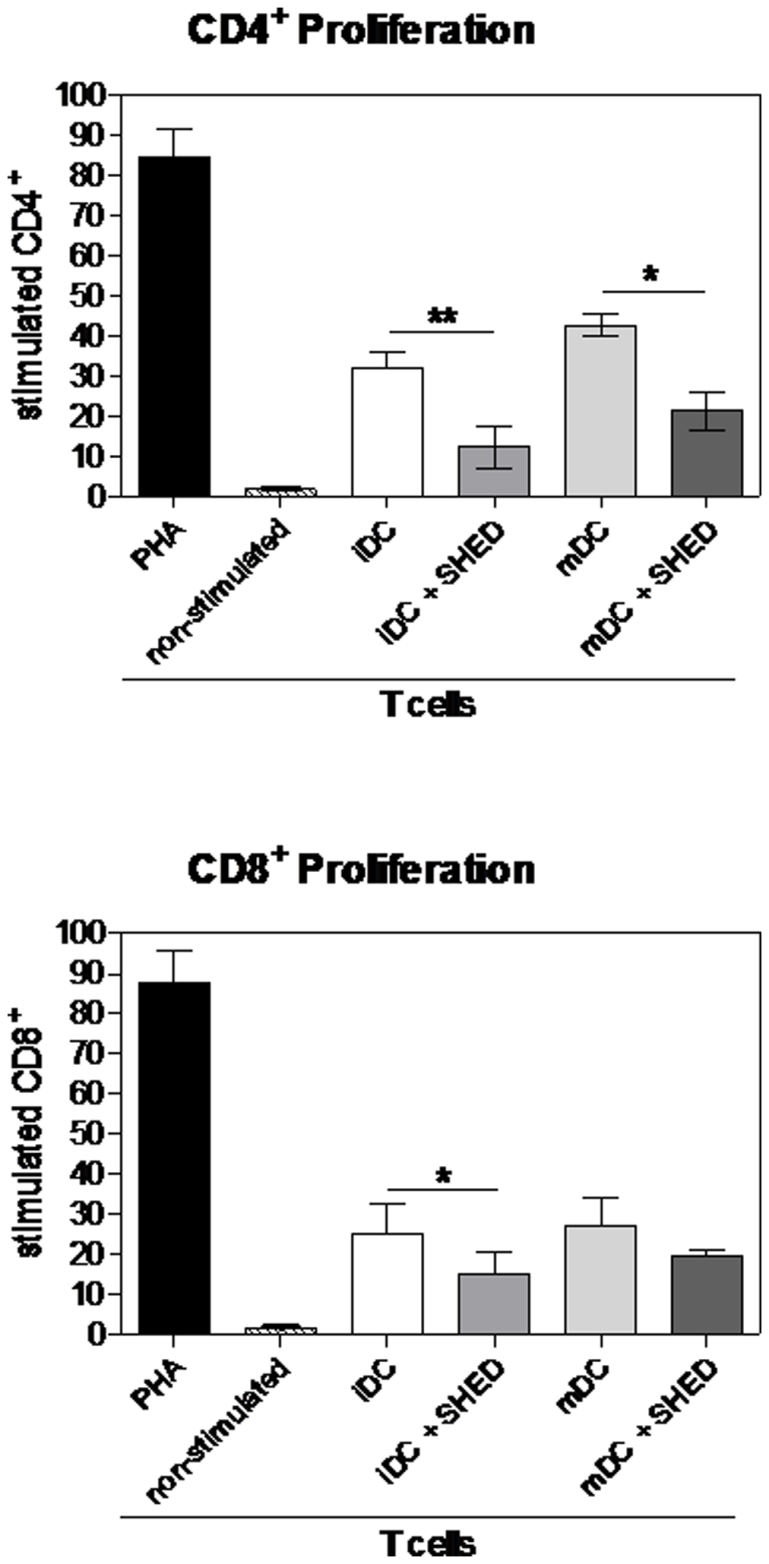
MoDCs previously stimulated by SHEDs are deficient to induce the proliferation of allogeneic T cells. Immature and mature moDCs co-cultured with SHEDs were isolated by anti-HLA-DR magnetic beads. MoDCs isolated were co-cultured with 1×10^5^ non-adherent cells CFSE stained (1∶10 ratio). After five days, T cells were stained with anti-CD4 and anti-CD8 antibodies, and CFSE dilution was determined by flow cytometry analysis. This analysis was previously performed within FSC and SSC gate, followed by CD4 or CD8 gate for T cells type of interest. PHA (Phytohemagglutinin A) was used as a polyclonal-positive stimulus. At least 20,000 events were acquired. iDC – without SHED contact; iDC+SHED – iDC previously co-cultured with SHED; mDC – without SHED contact; mDC+SHED – mDC previously co-cultured with SHED. Comparisons were done using t-test between iDC and iDC+SHED and between mDC and mDC+SHED co-cultured with T cells; *p≤0.05 and **p≤0.01 for the groups that showed significant differences; n = 3.

The rate of CD4^+^ lymphocytes proliferation, in presence of iDCs previously co-cultured with SHEDs, decreased 63% in comparison to iDCs not co-cultured with SHEDs (p≤0.01; Figure 6). In addition, mDCs previously co-cultured with SHEDs decreased their CD4^+^ T lymphocytes stimulation potential by circa 50% relative to mDCs differentiated without contact with SHEDs (p≤0.05). In the same way, the proliferation of CD8^+^ T lymphocytes was decreased by 40% (p≤0.05) and 26% (not significant) in co-culture with iDCs and mDCs differentiated in the presence of SHEDs, respectively (Figure 6). These findings show that DCs pre-incubated with SHEDs have their capability to stimulate T cell proliferation reduced.

**Figure 7:**
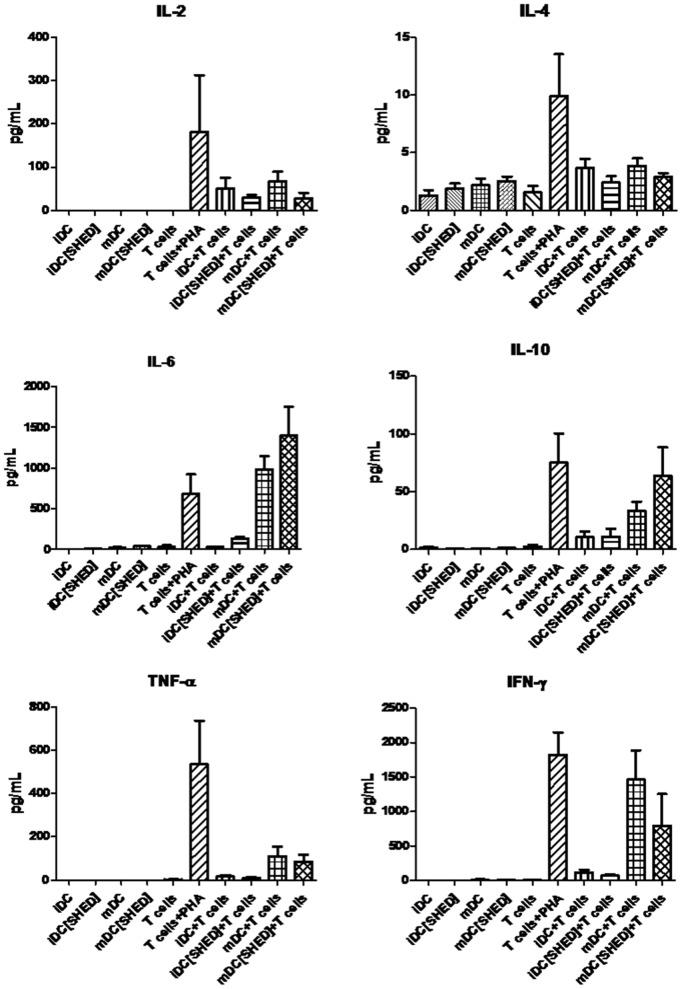
MoDCs pre-stimulated with SHEDs induce immune modulation on cytokines production in co-culture with T cells. Graphics indicate soluble factors levels (pg/mL; detection by CBA technique) present in moDCs culture previously treated with SHEDs (in a 1∶10 ratio; without T cells); and in the co-culture of allogeneic T cells with moDCs (previously co-cultured with or without SHEDs; both cultures in a 1∶10 ratio); both stimulated or not with LPS. Comparisons were done using t-test between iDC and iDC+SHED and between mDC and mDC+SHED; *p≤0.05 and **p≤0.01 for the groups that showed significant differences; n = 4.

Also, the cytokine profile of these co-cultures was determined (Figure 7). Interestingly, though not achieving statistical significance, a shift in the cytokine production in the co-cultures with SHED-exposed moDCs is discernible. Among the cytokines studied, there was a decrease in stimulatory cytokine levels (IL-2, IL-4, TNF-α and IFN-γ) and an increase in cytokines associated with the induction of regulatory responses (IL-6 and IL-10), a phenomenon that was more intense in the co-cultures with mDCs, but also detectable in the cultures with iDCs. IL-17 was not detected in any condition (data not shown).

### MoDCs Pre-incubated by SHEDs Increase the Frequency of FoxP3^+^IL-10^+^ among Responding T cells

**Figure 8:**
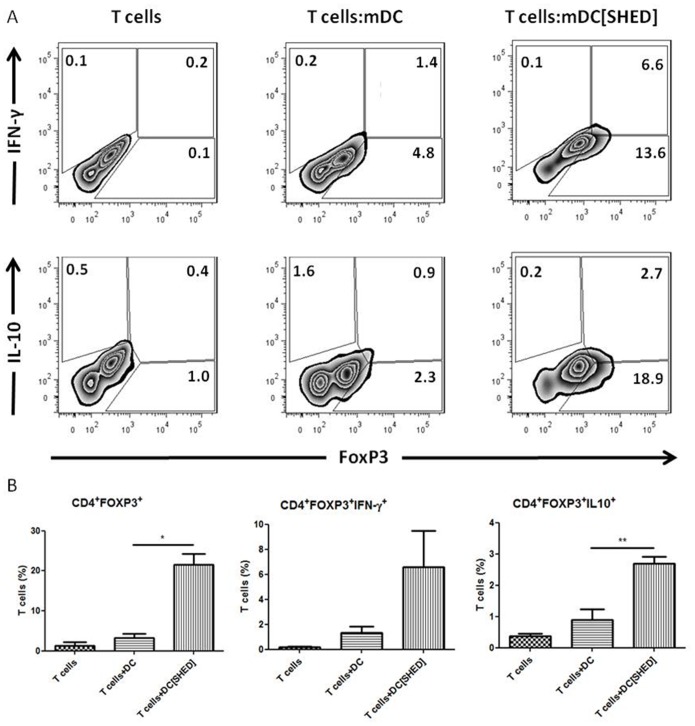
Increase in proportion of CD4^+^Foxp3^+^T cells and intracellular production of IL-10 or IFN-γ. MoDCs differentiated and matured in presence of SHEDs induce increase in proportion of CD4+Foxp3+ T cells and intracellular production of IL-10 or IFN-γ. CD4^+^ T cells expressing FoxP3 and IL-10 or IFN-y were analyzed after mDCs co-cultures (in the presence or not of SHEDs). (A) Representative average of population proportion CD4^+^FoxP3^+^IL-10^+^ or CD4^+^FoxP3^+^IFN-γ^+^ T cells. (B) Comparison between the groups means CD4^+^FoxP3^+^ (left), CD4^+^FoxP3^+^IFN-γ^+^ (center) and CD4^+^FoxP3^+^IL-10^+^ (right). Analysis was performed with four unrelated donors (n = 4). It was realized t-test between T cells+mDC and T cells+mDC[SHED] groups; *p≤0,05 and **p≤0,01 01 for the groups that showed significant differences.

Since the moDCs differentiated in the presence of SHEDs induced a decreased T cell proliferation and a cytokine profile that is associated with regulatory responses, the next step was to determine whether this putative “regulatory DCs” did, indeed, modulate the CD4^+^ T cell response by activating or increasing the frequency of FoxP3^+^ regulatory T cells. Accordingly, moDCs differentiated in the presence of SHEDs induced a substantial increase (seven fold) in the proportion of CD4^+^FoxP3^+^ T cells (p≤0.05) among the responding T cell population. Furthermore, these CD4^+^FoxP3^+^ T cells showed a significant increase in intracellular staining for IL-10 (p≤0.01; Figure 8), when compared to T cells stimulated by control moDCs.

## Discussion

MSCs are known to exhibit regulatory properties on several kinds of immune cells, however, in which extension SHEDs share this regulatory activity has been insufficiently investigated. We show here that, indeed, SHEDs have an effect upon moDCs that is in agreement with such an immunoregulatory role, thus opening the possibility of their eventual use for the control of immune-mediated conditions. DCs are key cells in immune responses, participating in several immune- and inflammatory-mediated diseases [5], [12]–[14]. *In vitro* studies have suggested that MSC inhibit IFN-γ and increase of IL-4 and IL-10 secretion [5], therefore favoring a better organ outcome in some animal models of Th1-mediated diseases [21].

Furthermore, MSCs can promote the down-modulation of several DC maturation markers and inhibit TNF-α secretion, leading to an immunological tolerance state, which can be beneficial for many diseases, for instance, chronic graft versus host disease [5]. These observations guided us to further explore the immune modulation capacity of a MSC isolated from exfoliated tooth, namely SHEDs, as a future promise for clinical therapeutic approaches.

First, we investigated if SHEDs would be affected in their phenotype, by culture under DCs differentiation medium. Our findings showed that SHEDs did not acquire myeloid (CD14) or DCs (CD11c and BDCA-1) markers, nor the HLA-DR molecule; furthermore, they maintained their multipotent stem cells markers (CD73, CD90 and CD105). Even though there are no works specifically related to SHEDs and immune modulation condition, our data corroborate with the result obtained by research groups that work with BMSCs that showed that these cells also were not immunogenic and were feasibly to use as immune regulatory proposes [3], [4], [7].

The next step was to explore if SHEDs influence moDCs differentiation and behavior. Blood monocytes were co-cultivated with SHEDs in conditions that induce DC differentiaton. In the presence of SHEDs, we observed a significant decrease of BDCA-1 (a specific marker for myeloid DCs type 1) and an increased level of CD14 molecule (monocyte marker) on immature DCs obtained after 7 days in culture, indicating that SHEDs hampered the differentiation of monocytes into DCs.

This observation is in agreement with data describing immune modulatory capacities of MSCs upon DCs. Zhang et al. (2004) showed that BMSCs inhibited the up-regulation of CD1a and inhibited the down-regulation of CD14 on DCs [4]. In addition, Lai et al. (2010) observed that BMSCs in co-culture with hematopoietic stem cells (HSCs) inhibited generation of many myeloid DCs subtypes, including CD14^+^ monocytes, CD4^+^ DCs (putative pan-DCs precursors), CD11c^+^Ecad^+^ Langerhans DCs (LCs), and CD11c^+^ myeloid DCs [14].

We further showed that iDCs generated in the presence of SHEDs have also a maturation deficit, since their stimulation with LPS yielded cells with lower CD40 expression. This effect could be a result by the reduction in their differentiation from monocytes once DCs stimulated with LPS also showed a significant decrease of BDCA-1, indicating an incomplete differentiation process. Again, this is compatible with results obtained by others, who observed inhibited expression of CD40, CD80 and CD86 molecules on mDCs surface when cocultivated with MSCs [4], [14], [20]. Likewise, Zhao et al. (2012) reached similar conclusions, observing that mDCs treated with BMSCs expressed higher levels of the myeloid lineage marker CD11b and were not able to increase the functional molecules, CD1a, CD80, CD83, CD86, and CD40 [22].

Many studies have showed that MSCs and DPSCs suppress DCs maturation and T cell proliferation (Le Blanc et al., 2003a; 2003b; Aggarwal et al., 2005; Chan et al., 2006; Pierdomenico et al. 2005), however, fewer groups have described the potential of MSCs to attenuate the propriety of DCs to stimulate T cells proliferation or to increase the ratio of CD4^+^FoxP3^+^ T cells among the responding population [4], [14], [22]. English et al. (2009) showed an increase in CD4^+^CD25^+^FoxP3^+^ T cells in co-cultures of BMSCs and CD4^+^ T cells, which was induced by soluble factors released by MSCs [23]. Similar results were obtained by Choi et al. (2012) that noted the induction of FoxP3^+^ Treg cells in co-cultures of mouse splenocytes, iDCs and BMSCs [20]; Zhao et al. (2012) showed that co-cultures of DCs, BMSCs and CD4^+^CD25^−^ T cells can generate CD4^+^CD25^+^FoxP3^+^ Treg [22]. Here, we show that also SHEDs present this ability to decrease DCs efficiency to stimulate CD4^+^ and CD8^+^ T lymphocytes. First, we observed that the proliferation rate of T cells stimulated by mDCs, differentiated and maturated in the presence of SHEDs, was reduced. Next, we demonstrate that among cells responding to these mature moDCs, the frequency of FoxP3^+^ T cells was significantly increased. Thus, our data support the model whereby MSC, specifically SHEDs, besides directly affecting T cell responses, can also affect these indirectly. This latter effect being dependent on the altered function of DCs differentiated in their presence, which have the propriety to increase CD4^+^FoxP3^+^ T cells generation.

It was postulated that CD4^+^FoxP3^+^ T cells are a source of IL-10 that may control exacerbated inflammatory responses [24]–[27]. Other authors indicate that Treg cells can produce IL-10 induced by their IL-10R in a feed-forward loop [28] and also, in certain infection contexts, they can express the T-bet transcription factor and produce IFN-y, without losing their suppressive ability [29]. Based on these reports, we investigated the production of IL10 and IFN-y by the CD4^+^FoxP3^+^ T lymphocytes stimulated by mDCs pre-treated with SHEDs. We found that, indeed, mDCs treated with SHEDs induce an increased proportion of CD4^+^FoxP3^+^IL-10^+^. Interestingly, though not reaching statistical significance, we also observed a tendency to an increased proportion of CD4^+^FoxP3^+^IFN-γ^+^ T cells among the responding cells.

These differences in T cell responses to the stimulus by SHEDs-exposed moDCs could be related to an altered cytokine secretion profile of the latter. Indeed, we observed a modulation in the pro- and anti-inflammatory cytokines present in moDCs-SHEDs co-cultures. Likewise, Zhao et al. (2012) showed in BMSCs-DCs co-culture, not only increased IL-10 and TGF-β1 secretion but also decreased IL-12 secretion; a pattern that was enhanced in the presence of LPS [22]. Aggarwal et al. (2005) observed that BMSCs in co-culture with LPS-activated myeloid type 1 DCs promoted TNF-α inhibition, while IL-10 secretion by plasmocytoid type 2 DCs was enhanced [5]. Other authors also described similar effects of BMSC, pointing to a modulation of the Th1/Th2 ratio [5], [20]. Thus, data from the literature and our results indicate that MSC, both derived from the bone marrow and from human exfoliated deciduous teeth, have the ability to increase anti-inflammatory cytokines, decrease pro-inflammatory cytokines and induce CD4^+^FoxP3^+^ T cells, all properties that could have relevant clinical applications in the modulation of disease conditions.

The immune response depends on the biology environment context. LPS, TNF-α, IFN-γ or CD40 ligand are signals that stimulate immune cells toward inflammatory immune response context. It is also worth noting that the immune suppressive/modulatory effects of SHEDs upon moDCs were not reversed by LPS. The context of MSCs immune modulation, as we showed, LPS used for DCs maturation enhanced immune modulatory SHEDs aspect. This is in agreement with the observations of Ryan et al. (2007), who showed that IFN-γ resulted in a significant increase in BMSCs production of HGF, IDO and TGF-β1 [30].

In conclusion, the present work shows that SHEDs affect monocytes differentiation into DCs, causing the generation of a “regulatory” profile on these moDCs cells. This is evidenced by the decrease of surface molecules related to DCs differentiation (BDCA1 and CD11c) and the maintenance of CD14 monocyte marker on immature cells. Phenotypic alterations persist even after LPS-induced maturation of the moDCs, which show reduction of CD40 activation marker, when differentiated in the presence of SHEDs. Furthermore, SHED-treated DCs displayed a reduced ability to expand T cells, and a bias toward the stimulation of CD4^+^FoxP3^+^IL-10^+^ T cells. This was accompanied by a modulation of the cytokine secretion pattern, with increase of anti-inflammatory and decrease of pro-inflammatory soluble factors in co-culture in the presence of SHEDs. These data reaffirm the MSCs immune modulatory potential and indicate that it is shared also by SHEDs. MSC, thus, through these properties, may play a role in homeostasis, helping to restore equilibrium to an environment disturbed by an “incoherent” immune response, resolving infections or injury [31]. Our data, shows that easily obtained MSCs, like SHEDs, keep this homeostatic role in experimental settings and could be, therefore, investigated and explored as potential tools for clinical immune therapy in autoimmune and inflammatory diseases.
